# Acceptance of healthy lifestyle nudges in the general population of Singapore

**DOI:** 10.1186/s12889-022-13668-x

**Published:** 2022-07-05

**Authors:** Yeow Wee Brian Tan, Edward Ryan Tan, Koh Yen Sin, P. V. AshaRani, Edimansyah Abdin, Kumarasan Roystonn, Peizhi Wang, Fiona Devi, Janhavi Vaingankar, Rob M van Dam, Chee Fang Sum, Eng Sing Lee, Wai Leng Chow, Siow Ann Chong, Mythily Subramaniam

**Affiliations:** 1 Research Division, Institute of Mental Health, 10 Buangkok View, Buangkok Green Medical Park, Singapore, 539747 Singapore; 2 Yong Loo Lin School of Medicine, 10 Medical Drive, Singapore, 117597 Singapore; 3 Saw Swee Hock School of Public Health, 12 Science Drive, Singapore, 117549 Singapore; 4 Admiralty Medical Centre, Khoo Teck Puat Hospital, 676 Woodlands Drive 71, Singapore, 730676 Singapore; 5 National Healthcare Group Polyclinics, 3 Fusionopolis Link. Nexus@One-North, Singapore, 138542 Singapore; 6 Epidemiology and Communicable Diseases Division, Ministry of Health, Singapore, 169854 Singapore

**Keywords:** Healthy lifestyle, Nudges, Acceptance, Singapore

## Abstract

**Background:**

In recent years, behaviourally driven policies such as nudges have been increasingly implemented to steer desired outcomes in public health. This study examines the different nudges and the socio-demographic characteristics and lifestyle behaviours that are associated with public acceptance of lifestyle nudges.

**Methods:**

The study used data from the nationwide Knowledge, Attitudes and Practices study (KAP) on diabetes in Singapore. Three types of nudges arranged in increasing order of intrusiveness were examined: (1) information government campaigns, (2) government mandated information and (3) default rules and choice architecture. Acceptance was assessed based upon how much respondents ‘agreed’ with related statements describing heathy lifestyle nudges. Multivariable linear regressions were performed with socio-demographics and lifestyle behaviours using scores calculated for each nudge.

**Results:**

The percentage of respondents who agreed to all statements related to each nudge were: 75.9% (information government campaigns), 73.0% (government mandated information), and 33.4% (default rules and choice architecture). Respondents of Malay/Others ethnicity (vs. Chinese) were more likely to accept information government campaigns. Respondents who were 18 – 34 years old (vs 65 years and above), female, of Malay/Indian ethnicity (vs Chinese), were sufficiently physically active, and with a healthier diet based on the DASH (Dietary Approach to Stop Hypertension) score were more likely to accept nudges related to government mandated information. Respondents of Malay/Indian ethnicity (vs Chinese), and who had a healthier diet were more likely to accept default rules and choice architecture.

**Conclusion:**

Individuals prefer less intrusive approaches for promoting healthy lifestyle. Ethnicity and lifestyle behaviours are associated with acceptance of nudges and should be taken into consideration during the formulation and implementation of behaviourally informed health policies.

**Supplementary Information:**

The online version contains supplementary material available at 10.1186/s12889-022-13668-x.

## Background

Leading a healthy lifestyle by engaging in behaviours such as healthy eating, and regular exercise are well-established contributors to good health and successful aging [1]. Nonetheless, developed nations such as Singapore have seen a marked rise in largely preventable chronic medical conditions such as hypertension, diabetes, high total cholesterol, and obesity [2]. Given the multitude of health benefits that adopting a healthy lifestyle confers, it is unsurprising that there has been greater focus directed towards promoting healthier lifestyle choices amongst citizens to curb the issue. In recent years, such efforts have shifted toward a more nuanced approach through the application of behavioural insights to influence decision making; a concept known as nudging [3, 4].

Nudging can be broadly defined as “any aspect of the choice architecture that alters people’s behaviour in a predictable way without forbidding any options, or significantly changing their economic incentives” [5]. Generally, nudges act as a low-cost, less intrusive method of public policy. While nudges have been widely used in the public domain, one area of interest is the usage of nudges as a mean of promoting healthier lifestyle choices [6, 7]. Examples of a health-nudge would relate to the replacement of unhealthy products (such as sweets) with healthier ones (protein bars) at supermarket checkouts so that people would select the healthier product instead. The influence on decision-making of such an approach is that it may potentially have a significant effect on public health without forcing anyone to commit to or do anything at all. A meta-analysis of 37 papers on the efficacy of nudge theory found that on average, nudges were successful in increasing nutritional choices by up to approximately 15.3% [8]. Given its effectiveness, there is an increasing global interest in testing and implementing nudges as a means of promoting healthy lifestyle [9, 10].

While nudges are generally effective, there exists a rich debate surrounding the use of nudges, with proponents maintaining that nudges do not reduce autonomy, but increase it in some cases while critics claiming they are manipulative [11]. Furthermore, some critics claim that nudges are used to achieve goals that are not particularly useful or helpful to the person or society [12]. Accordingly, current literature provides further evidence highlighting the disparity in citizens’ views and endorsement of nudges across various nations. For example, Sunstein et al. [13] reported markedly high approval ratings in Asian countries such as China and South Korea. Surveying 952 people in Sweden and the United States, Hagman et al. [14] reported that strong majorities in both countries were in favour of a wide variety of nudges. Similarly, Krisam et al. [15] reported a strong majority of German citizens endorsing nudges as an accepted method to promote health behaviours. Conversely, countries such as Hungary, Denmark and Japan reported relatively low scores of approvals [13]. Specifically, while the majority in these nations do tend to approve of the tested nudges, the levels of approval are consistently low, and in some cases, approval rates fall below 50% [16]. Owing to this disparity, it follows that determining the public’s perception towards nudges is an important precursor to the implementation of any form of nudge. Regardless of the type of intervention, public acceptance is considered to be one of three key aspects that should be taken into consideration prior to implementation [17]. As reported in prior studies, public acceptance can play a defining role in the effectiveness of the nudge implemented to the extent that in some cases, such impact can be observed even when the majority of a population does not know of nudging [15, 18]. Essentially, the evidences highlights that public acceptance can serve as a form of permission slip, whereby either widespread approval or disapproval can determine a predicted outcome which may serve to guide policy makers in their decision-making process [16].

The aforementioned studies present valuable insights exploring public attitudes toward nudges across various nations. Yet, there remains relatively little work exploring the approval rates of nudges in the domain of healthy lifestyle within a multi-ethnic population like Singapore. Singapore is a multi-ethnic city-state situated in Southeast Asia with a population of approximately 5.6 million of which 4.1 million are Singapore residents (Singapore citizens or permanent residents) [19]. The population largely comprises inhabitants from three major Asian ethnic groups: Chinese (76.0%), Malay (15.0%) and Indian (7.5%) [20]. Given its diverse ethnic composition, a study in this setting provides a unique opportunity to elucidate acceptance towards healthy lifestyle nudges within a multi-ethnic population.

To address the gaps in current literature, the present study aims to: 1) investigate the levels of approval regarding healthy lifestyle nudges, and 2) identify socio-demographics and lifestyle behaviours (sedentary behaviour, physical activity, and dietary patterns) that are associated with acceptance of healthy lifestyle nudges.

## Method

### Participants and procedures

 The data for this research comes from a population based, cross-sectional study aimed at evaluating the Knowledge, Practices and Attitudes towards Diabetes Mellitus (DM) amongst residents of Singapore aged 18 years and above. A more detailed methodology of the study can be found in an earlier paper [21]. Briefly, the sample was randomly selected via a disproportionate stratified sampling design according to ethnicity (Chinese, Malay, Indian, Others) and age groups (18–34, 35–49, 50–64, 65 and above) from a national population registry database of all citizens and permanent residents within Singapore. The study oversampled certain minority populations, such as Malay and Indian ethnicities, as well as those above 65 years of age, in order to improve the reliability of the parameter estimates for these subgroups.

Citizens and permanent residents who were randomly selected were sent notification letters followed by home visits by trained interviewers from a survey research company to obtain their informed consent to participate in the study. Face-to-face interviews with those who were agreeable to participate were conducted in their preferred language (English, Mandarin, Malay, or Tamil). Responses were captured using computer assisted personal interviewing. Individuals who were unable to be contacted due to incomplete or incorrect addresses, or living outside of the country, or were incapable of attending the interview due to severe physical or mental conditions, language barriers, or were institutionalised or hospitalised at the time of the survey were excluded from the study. For those aged 18 to 20 years, parental consent was sought as the official age of majority in Singapore is 21 years and above. The study closed recruitment with a final response rate (total completed interview / [total number of sample – eligible cases]) of 66.2%.

### Measures

#### Healthy lifestyle nudges questionnaire

The survey questionnaire built upon prior work limited to Europe [16]. The version included a total of 15 items. To adjust to the Singapore context, this number was reduced to 8. The selection was categorised into three groups in terms of increasing intrusiveness: i) information government campaigns: purely government campaigns to educate individuals about healthy lifestyle choices ii) government mandated information: mandatory information nudges imposed by government requiring disclosure of nutritional value and health risk of food e.g. calorie labels in restaurants, high salt content warnings, nutritional traffic lights and iii) default rules and choice architecture for retailers to support healthy foods e.g. sweet-free cashier zones. Items were administered via a 5-point Likert scale ranging from 1 = “Strong Agree” to 5 = “Strongly Disagree”.

#### Chronic physical conditions

A modified version of the World Mental Health Composite International Diagnostic Interview (CIDI) version 3.0 checklist of chronic medical conditions was used, and the respondents were asked to report any of the conditions listed in the checklist [22]. The question was read as, “I am going to read to you a list of health problems some people have. Has a doctor ever told you that you have any of the following chronic medical conditions?” This was followed by a list of 18 chronic physical conditions (such as asthma, high blood sugar, hypertension, arthritis, cancer, neurological condition, Parkinson’s disease, stroke, congestive heart failure, heart disease, back problems, stomach ulcer, chronic inflamed bowel, thyroid disease, kidney failure, migraine headaches, chronic lung disease, and hyperlipidaemia) which are prevalent among Singapore’s population.

#### Physical activity and sedentary behaviour

The Global Physical Activity Questionnaire (GPAQ) is a 16-item instrument developed by the World Health Organisation to measure physical activity [23]. Translations of the GPAQ to Mandarin, Malay and English were permitted by the publisher. Respondents were asked about the duration and frequency of vigorous and moderate intensity activities for work, transport, or leisure during a typical week. Utilising this information, the GPAQ scoring protocol allows for the calculation of weekly metabolic equivalents of tasks (MET) values, with one MET being equivalent to the caloric consumption of 1 kcal/kg/hour. MET values were calculated by multiplying weekly vigorous activity minutes by 8 and moderate-intensity minutes by 4, and a cut-off was applied following recommendations in the GPAQ analysis guide to dichotomise physical activity [24]. Those who met the following criteria for physical activity for work, during transport and leisure time throughout the week were classified as “sufficiently active”:

i) At least 150 min of moderate-intensity physical activity OR
ii) 75 min of vigorous-intensity physical activity OR
iii) An equivalent combination of moderate- and vigorous-intensity physical activity achieving at least 600 MET-minutes per week.

Individuals who did not meet the above criteria were classified as “insufficiently active”.

The GPAQ also contains a single item: “How much time do you usually spend sitting or reclining on a typical day?”, which was used as a measure of sedentary behaviour. Based on two meta-analyses by Chau et al. & Ku et al. [25, 26], ≥ 7-h/day cut-off was utilised to differentiate between levels of self-reported sedentary behaviour.

#### Diet screener

The diet screener comprises a list of 30 food/beverage items, that respondents rate on a 10-point scale ranging from ‘never/rarely’ to ‘6 or more times per day’, the frequency at which they consumed a particular food/beverage within the last one year [27]. The diet screener was interviewer-administered. Standard serving sizes were indicated for each food/beverage item to facilitate this process. Intake frequencies were standardised to a number of servings per day for each food/beverage item. DASH scores were calculated to account for seven intake components: fruit, vegetables, nuts/legumes, whole grains, red and processed meat, low fat dairy, and sweetened beverages. For each of these seven components, participants received a score between 1 and 5 corresponding to the quintile of the intake they fall in, with reverse scoring utilised for meat and sweetened beverages, and these seven quintile scores were summed to form the overall DASH score.

#### Socio-demographic information and body mass index

Socio-demographic data on age (18–34, 35–49, 50–64 and 65 and above), sex (Female, Male), ethnicity (Chinese, Malay, Indian and Others), education (Primary and below, Secondary, Pre-U/Junior College, Vocational Institute/ITE, Diploma, Degree, professional certifications and above), marital status (Single, Married/Cohabiting, Divorced/Separated/Widowed), employment (Employed, Economically inactive and Unemployed), and monthly personal income in SGD (Below $2,000, $2,000-$3,999, $4,000-$5,999, $6000-$9,999 and $10,000 and above, and no income) were collected. Further, Body Mass Index (BMI) scores were categorised into four groups based on World Health Organisation guidelines: ‘underweight (< 18.5 kg/m^2^), ‘normal range’ (≥ 18.5 kg/m^2^ and < 25 kg/m^2^), ‘overweight’ (≥ 25 kg/m^2^ and < 30 kg/m^2^), and ‘obese’ (> 30 kg/m^2^) [28].

### Statistical analysis

Survey weights were included in the analysis to account for disproportionate stratified sampling design. The final weights were determined using sampling design weights, non-response adjustment weights and post-stratification adjustment weights. The post-stratification adjustment weights were constructed using ethnicity and age. Unweighted frequencies and weighted percentages were presented for each of the 8-items in the healthy lifestyle nudge questionnaire. To provide the unweighted frequencies and weighted percentages for the acceptance of each nudge, the responses were classified based on the number of related items that the respondents ‘Agreed’ to; the definition of ‘Agreed’ being the indication of either ‘Strongly agree’ or ‘Agree’ for each related item. In addition, the degree of acceptance for each nudge was stratified based on the number of chronic conditions: (i) no chronic condition, (ii) one chronic condition, and (iii) two or more chronic conditions.

To examine the significant correlates of acceptance for each nudge, the responses from the items were reverse coded. Following which, the rating for the related items were added up to obtain a score for each nudge, with higher score indicating greater acceptance to the specific nudge. Using the scores as the outcome variables, multivariable linear regression was performed for each nudge with the following independent variables: age, sex, education, marital status, employment, monthly personal income, BMI, physical activity, sedentary behaviour, and DASH score. Standard errors and significance tests were adjusted for survey weights using Taylor series’ linearisation method. The above analysis was conducted using STATA/SE 17.0 (College Station, Texas), with two-tailed tests assuming 5% significance level.

## Results

### Sociodemographic characteristics

In total, 2895 respondents participated in the survey. All age groups were sufficiently represented, with most of the respondents (29.9%) belonging to the 18 to 34 years old age group and least (15.1%) from the 65 years and older age group. Participants of both male and female sexes were represented equally. For BMI, 55.7% of respondents were in the normal range, with 7.2% in the underweight, 27.7% in the overweight, and 9.4% in the obese categories respectively. 45.9% of respondents did not have any chronic condition, while 26.4% had one chronic condition, and 27.7% had two or more chronic conditions (Table 1).

**Table 1:** Sociodemographic characteristics of overall sample

	**Weighted %**	**Unweighted %**	**n**
**Age groups (years)**			
18 to 34	29.9%	28.4%	823
35 to 49	28.2%	24.8%	719
50 to 64	26.8%	26.7%	774
65 and above	15.1%	20.0%	579
**Sex**			
Female	51.6%	50.9%	1474
Male	48.4%	49.1%	1421
**Ethnicity**			
Chinese	75.8%	27.5%	796
Malay	12.7%	33.6%	974
Indian	8.6%	31.7%	918
Others	2.9%	7.2%	207
**Education**			
Primary and below	20.4%	22.0%	637
Secondary	20.3%	23.6%	684
Pre-U/Junior College	4.8%	4.4%	126
Vocational Institute/ITE	6.6%	9.2%	267
Diploma	18.5%	16.6%	479
Degree, professional certification, and above	29.5%	24.3%	702
**Marital Status**			
Married/Cohabiting	61.7%	64.3%	1860
Single	29.2%	25.3%	731
Separated/Widowed/Divorced	9.1%	10.5%	303
**Employment status**			
Employed	70.5%	66.8%	1933
Economically Inactive	25.4%	28.6%	829
Unemployment	4.1%	4.6%	133
**Monthly income (Personal)**			
Below 2,000	40.1%	44.6%	1236
2,000 to 3,999	25.1%	25.2%	698
4,000 to 5,999	13.4%	11.5%	318
6,000 to 9,999	8.2%	6.6%	183
10,000 and above	5.9%	4.2%	117
No income	7.3%	7.9%	219
**BMI**			
Normal range ≥ 18.5 and < 25	55.7%	46.9%	1263
Underweight < 18.5	7.2%	5.6%	151
Overweight ≥ 25 and < 30	27.7%	31.9%	858
Obese ≥ 30	9.4%	15.6%	420
**Number of chronic conditions**			
No chronic condition	45.9%	42.6%	1229
One chronic condition	26.4%	26.5%	766
At least two or more chronic conditions	27.7%	30.9%	892
**Physical activity**			
Sufficiently active (≥ 600 MET-minutes per week)	83.4%	84.0%	2429
Insufficiently active (< 600 MET-minutes per week)	16.6%	16.0%	464
**Sedentary behaviour**			
< 7 h/day	52.2%	54.5%	1577
≥ 7 h/day	47.8%	45.5%	1317
**DASH Score**			
< 16	30.7%	25.8%	772
16 to ≤ 19	26.2%	23.5%	703
19 to ≤ 22	20.0%	20.2%	606
> 22	23.1%	30.5%	814

^a^Institute of Technical Education

### Approval of healthy lifestyle nudges

#### Information government campaigns

**Fig. 1:**
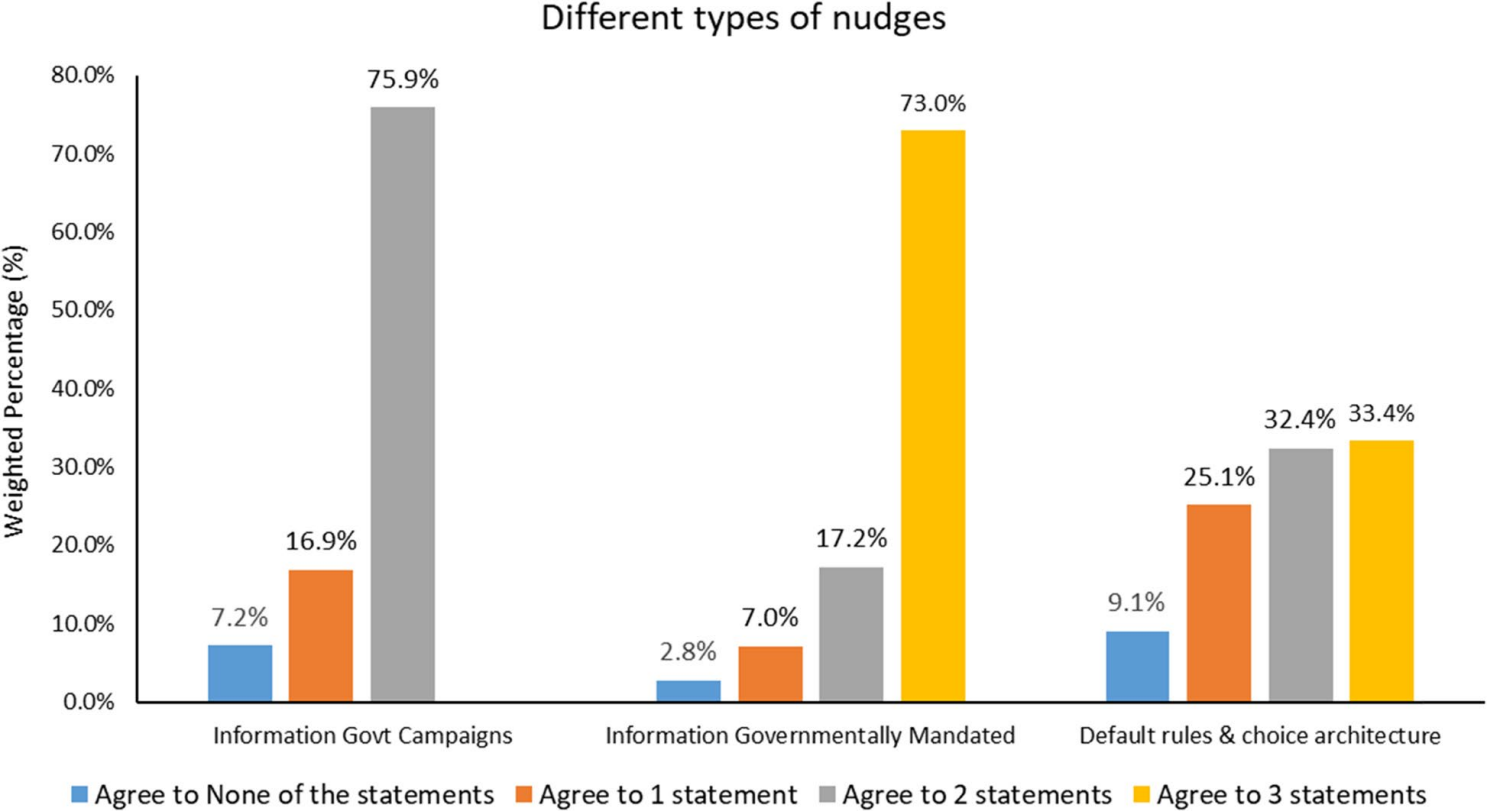
Breakdown in percentage of individuals who agree to statements within the nudge classifications of information government campaigns, information governmentally mandated, and default rules and choice architecture

**Fig. 2:**
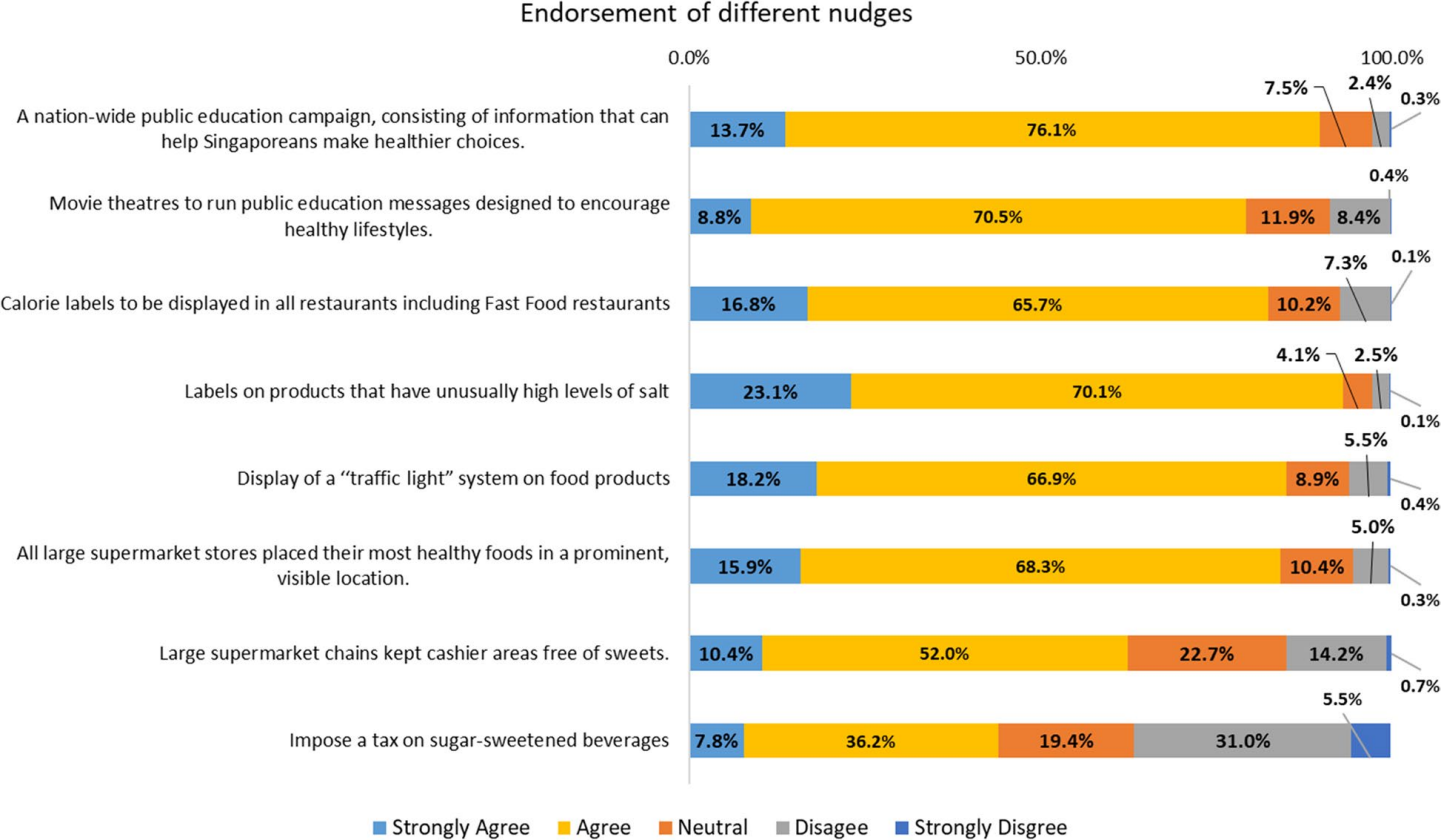
Breakdown in percentage of individuals’ approval regarding each nudge statement

This category of information government campaigns contains the least intrusive of the nudges in that they involved mere information provision by the government; (1) public education campaigns to help Singaporeans make healthier choices and (2) similar public campaigns in movie theatres to encourage healthy lifestyles. Majority expressed their approval for nudges in this category; with 75.9% agreeing to both statements, 16.9% agreeing to at least one statement, while 7.2% were not in agreement with any (Fig. 1). Specifically, 89.8% strongly endorsed or endorsed public education campaigns to help Singaporeans make healthier choices, and 79.3% strongly endorsed or endorsed similar public campaigns in movie theatres to encourage healthy lifestyles (Fig. 2). Overall, the average approval rating for information government campaigns was 7.7 (SD = 1.2) (Table 2).

**Table 2:** Mean (SD) of policy acceptance based on nudge classifications

	**Weighted Mean (SD)**	**Unweighted Mean (SD)**
Information government campaigns^a^	7.7 (1.2)	7.9 (1.1)
Information government mandated^b^	12.0 (1.7)	12.1 (1.7)
Default rules and choice architecture^c^	10.6 (2.0)	10.8 (2.0)

^a^ Missing observation: Information government campaigns (*n* = 10)

^b^ Missing observation: Information government mandated (*n* = 12)

^c^ Missing observation: Default rules and choice architectures (*n* = 5)

#### Government mandated information

Three nudges designed to promote healthier choices grouped in this category included: (3) calorie labels to be displayed in restaurants (4) salt labels (for products with particularly high salt content levels), and (5) a “traffic light” system to indicate more or less healthy food products. Given that such nudges require the action of private institutions, it might be perceived as more intrusive in comparison to information government campaigns. Nonetheless, majority expressed their approval for nudges in this category; with 73.0% agreeing to all three statements, 17.2% agreeing to two statements, 7.0% agreeing to one statement, while 2.8% were not in agreement with any (Fig. 1). Specifically, 82.5% strongly endorsed or endorsed calorie labels, 93.2% strongly endorsed or endorsed salt labels (for products with particularly high salt content levels), and 85.1% strongly endorsed or endorsed a “traffic light” system to indicate more or less healthy food products (Fig. 2). Overall, the average approval rating for information government mandated was 12.0 (SD = 1.7) (Table 2).

#### Default rules & choice architecture

Of the three categories, default rules and choice architecture are often the most prominent and effective nudges and seemingly the most intrusive from the categories listed. The statements grouped under this category were: (6) placing healthier food products in prominent or more visible location, (7) sweet-free cashier zones, and (8) imposing tax on sugar-sweetened beverages. In total, 33.4% agreed with all three statements, 32.4% agreed with two statements, 25.1% agreed with only one statement, while 9.1% were not in agreement with any (Fig. 1). Specifically, 84.2% strongly endorsed or endorsed placing healthier food products in prominent or more visible location, 62.4% strongly endorsed or endorsed sweet-free cashier zones, and 44.0% strongly endorsed or endorsed imposing tax on sugar-sweetened beverages (Fig. 2). Overall, the average approval rating for default rules and choice architecture was 10.6 (SD = 2.0) (Table 2).

### Approval of healthy lifestyle nudges as stratified by chronic conditions

Table 3 summarises the approval of healthy lifestyle nudges according to the three categories as stratified by the number of chronic conditions per individual: i) no chronic condition, ii) one chronic condition, and iii) two or more chronic conditions. Additionally, the weighted and unweighted means and standard deviations are provided across the categories for comparisons. For information government campaigns, majority expressed their approval across all three chronic conditions subgroups. Within the study population, 74.9% of individuals with no chronic condition, 74.3% of individuals with one chronic condition, and 79.2% of individuals with two or more chronic conditions agreed with all statements classified under information government campaigns.

**Table 3:** Percentage of individuals who agree to 0/1/2/3 statements, weighted and unweighted mean and standard deviation as stratified by number of chronic conditions

**Information Government Campaigns **(*p*-value = 0.358) ^^^						
	**Agree to None of the statements [% (n)]**	**Agree to 1 statement [% (n)]**	**Agree to 2 statements [% (n)]**		**Weighted Mean (SD)**	**Unweighted Mean (SD)**
**Chronic illness**						
No chronic illness	8.3% (76)	16.8% (193)	74.9% (958)		7.7 (1.1)	7.9 (1.1)
One chronic illness	6.7% (46)	18.9% (129)	74.3% (591)		7.8 (1.2)	7.8 (1.1)
At least two or more chronic illness	5.6% (38)	15.2% (123)	79.2% (727)		7.9 (1.2)	7.9 (1.1)
**Government Mandated Information **(*p*-value = 0.069) ^^^						
	**Agree to None of the statements [% (n)]**	**Agree to 1 statement [% (n)]**	**Agree to 2 statements [% (n)]**	**Agree to 3 statements [% (n)]**	**Weighted Mean (SD)**	**Unweighted Mean (SD)**
**Chronic illness**						
No chronic illness	3.1% (33)	7.4% (77)	20.0% (226)	69.5% (892)	11.9 (1.6)	12.1 (1.7)
One chronic illness	2.0% (22)	7.6% (45)	17.0% (125)	73.4% (574)	12.1 (1.7)	12.1 (1.7)
At least two or more chronic illness	2.9% (18)	6.0% (51)	12.4% (111)	78.7% (705)	12.0 (1.8)	12.0 (1.7)
**Default Rules & Choice Architecture** (*p*-value = 0.444) ^^^						
	**Agree to None of the statements [% (n)]**	**Agree to 1 statement [% (n)]**	**Agree to 2 statements [% (n)]**	**Agree to 3 statements [% (n)]**	**Weighted Mean (SD)**	**Unweighted Mean (SD)**
**Chronic Conditions**						
No chronic conditions	10.2% (95)	24.8% (278)	34.0% (420)	31.0% (436)	10.5 (1.9)	10.7 (2.0)
One chronic condition	8.8% (50)	26.5% (174)	31.1% (251)	33.7% (291)	10.6 (2.1)	10.8 (2.0)
At least two or more chronic conditions	7.5% (48)	24.3% (177)	31.0% (285)	37.3% (381)	10.6 (2.1)	10.8 (1.9)

^^^*p*-values obtained based on Rao-Scott corrected Chi-square statistics

For government mandated information, a similar pattern was observed whereby majority expressed their approval across all three chronic conditions subgroups. Specifically, 69.5% of individuals with no chronic condition, 73.4% with one chronic condition, and 78.7% with two or more chronic conditions agreed with all statements classified under government mandated information.

For default rules and choice architecture, results were mixed as fewer expressed approval across all three chronic conditions subgroups. In total, 31.0% of individuals with no chronic condition, 33.7% of individuals with one chronic condition, and 37.3% of individuals with two or more chronic conditions agreed with all statements classified under default rules and choice architecture.

### Socio-demographic and lifestyle correlates of health-nudges

#### Information government campaigns

Results examining the socio-demographic and lifestyle behaviours correlates of healthy lifestyle nudges can be found in Table 4. Additionally, the differences between the groups of individuals for each outcome can be found in Supplementary Table 1. For information government campaigns, ethnicity was significantly associated with greater approval of nudges classified in this category. Individuals of Malay (B = 0.23, *p* = 0.001) and Others (B = 0.32, *p* < 0.001) ethnicities reported significantly greater approval as compared to those of Chinese ethnicity.

**Table 4:** Results of multivariable linear regression analyses examining the socio-demographic and lifestyle correlates of health-nudges

	**Information Government Campaigns**				**Information Governmentally Mandated**				**Default Rules & Choice Architecture**			
	**Beta**	**95% CI**		***P*-value**	**Beta**	**95% CI**		***P*-value**	**Beta**	**95% CI**		***P*-value**
**Age groups (years)**												
18 to 34 (Reference)												
35 to 49	0.05	-0.18	0.27	0.692	-0.29	-0.61	0.02	0.066	-0.10	-0.46	0.26	0.581
50 to 64	0.19	-0.08	0.45	0.163	-0.35	-0.72	0.01	0.058	-0.06	-0.48	0.36	0.777
65 and above	0.13	-0.15	0.42	0.363	-0.45	-0.87	-0.03	**0.035**	-0.01	-0.51	0.50	0.974
**Sex**												
Female (Reference)												
Male	-0.01	-0.14	0.13	0.898	-0.26	-0.45	-0.07	**0.008**	-0.14	-0.38	0.10	0.240
**Ethnicity**												
Chinese (Reference)												
Malay	0.23	0.10	0.36	** < 0.001**	0.21	0.00	0.41	**0.047**	0.39	0.15	0.62	**0.001**
Indian	0.13	0.00	0.27	0.051	0.39	0.20	0.59	** < 0.001**	0.49	0.26	0.72	** < 0.001**
Others	0.32	0.14	0.50	** < 0.001**	0.29	-0.03	0.60	0.073	0.30	-0.09	0.68	0.128
**Education**												
Primary and below (Reference)												
Secondary	-0.01	-0.20	0.17	0.902	-0.18	-0.47	0.12	0.238	-0.13	-0.48	0.22	0.457
Pre-U/Junior College	0.01	-0.32	0.33	0.976	0.23	-0.29	0.75	0.384	0.04	-0.58	0.66	0.903
Vocational Institute/ITE	0.08	-0.21	0.38	0.586	-0.14	-0.60	0.33	0.570	0.09	-0.41	0.60	0.718
Diploma	0.18	-0.05	0.41	0.123	0.28	-0.08	0.64	0.125	0.13	-0.30	0.56	0.556
Degree, professional certification, and above	0.11	-0.15	0.37	0.411	-0.04	-0.42	0.35	0.854	0.11	-0.33	0.56	0.617
**Marital Status**												
Married/Cohabiting (Reference)												
Single	-0.12	-0.32	0.08	0.230	-0.25	-0.55	0.04	0.090	-0.31	-0.64	0.02	0.062
Separated/Widowed/Divorced	-0.02	-0.23	0.19	0.876	0.07	-0.21	0.36	0.607	-0.10	-0.47	0.26	0.579
**Employment status**												
Employed (Reference)												
Economically Inactive	0.06	-0.11	0.23	0.506	0.10	-0.16	0.37	0.452	0.25	-0.07	0.57	0.129
Unemployment	0.06	-0.24	0.35	0.704	0.04	-0.42	0.51	0.860	0.16	-0.41	0.72	0.586
**Monthly income (Personal)**												
Below 2,000 (Reference)												
2,000 to 3,999	-0.01	-0.19	0.17	0.890	-0.09	-0.34	0.17	0.510	-0.20	-0.50	0.11	0.205
4,000 to 5,999	-0.21	-0.46	0.04	0.103	-0.07	-0.38	0.24	0.661	-0.09	-0.52	0.34	0.671
6,000 to 9,999	-0.18	-0.52	0.16	0.293	0.01	-0.41	0.43	0.971	0.20	-0.33	0.73	0.460
10,000 and above	-0.26	-0.63	0.11	0.175	0.23	-0.34	0.81	0.425	0.30	-0.35	0.94	0.364
No income	-0.04	-0.31	0.24	0.780	-0.15	-0.57	0.28	0.499	-0.14	-0.61	0.32	0.539
**BMI**												
Normal range (Reference)												
Underweight	-0.29	-0.60	0.03	0.078	-0.14	-0.59	0.31	0.536	-0.14	-0.59	0.31	0.537
Overweight	0.06	-0.10	0.21	0.479	-0.12	-0.34	0.09	0.272	-0.01	-0.28	0.26	0.941
Obese	-0.12	-0.34	0.09	0.267	-0.30	-0.60	0.01	0.060	-0.25	-0.64	0.13	0.193
**Number of chronic conditions**												
No chronic illness (Reference)												
One chronic illness	-0.08	-0.25	0.08	0.310	0.10	-0.13	0.33	0.405	-0.04	-0.31	0.23	0.758
At least two or more chronic illness	0.00	-0.17	0.17	0.994	0.19	-0.07	0.45	0.148	-0.04	-0.34	0.26	0.789
**Physical activity**												
Sufficiently active (Reference)												
Insufficiently active	0.04	-0.13	0.20	0.643	-0.40	0.13	0.67	**0.004**	0.22	-0.08	0.53	0.147
**Sedentary behaviour**												
< 7 h/day (Reference)												
≥ 7 h/day	-0.14	-0.27	0.00	0.470	-0.08	-0.28	0.12	0.436	0.00	-0.24	0.24	0.992
**DASH Score**												
< 16 (Reference)												
16 to ≤ 19	0.13	-0.05	0.30	0.157	0.16	-0.10	0.43	0.233	0.28	-0.02	0.58	0.067
19 to ≤ 22	0.18	-0.01	0.36	0.066	0.31	0.03	0.59	**0.028**	0.52	0.18	0.86	**0.003**
> 22	0.07	-0.11	0.26	0.438	0.35	0.10	0.60	**0.006**	0.56	0.25	0.88	** < 0.001**

^a^Institute of Technical Education

#### Government mandated information

For government mandated information, age, sex, ethnicity, physical activity, and DASH score were significantly associated with greater approval of nudges. Individuals who were 65 years old and above (B = -0.45, *p* = 0.035) were significantly associated with lower approval as compared to those who were 18 – 34 years old. Males (B = -0.26, *p* = 0.008) reported significantly lower approval as compared to females. Individuals of Malay (B = 0.21, *p* = 0.047) and Indian (B = 0.39, *p* < 0.001) ethnicities reported significantly greater approval as compared to those of Chinese ethnicity. Insufficient physical activity (B = -0.40, *p* = 0.004) had a significantly lower approval as compared to those with sufficient physical activity. Individuals with DASH score 19 to ≤ 22 (B = 0.31, *p* = 0.028) and > 22 (B = 0.35, *p* = 0.006) reported significantly greater approval as compared to individuals with DASH score < 19.

#### Default rules & choice architecture

For default rules and choice architecture, ethnicity and DASH score were significantly associated with greater approval of nudges. Individuals of Malay (B = 0.39, *p* = 0.001) and Indian (B = 0.49, *p* < 0.001) ethnicities reported significantly greater approval as compared to those of Chinese ethnicity. Individuals with DASH score 19 to ≤ 22 (B = 0.52, *p* = 0.003) and > 22 (B = 0.56, *p* < 0.001) reported significantly greater approval as compared to individuals with DASH score < 19.

## Discussion

### Approval rates across the population

Overall, the results demonstrated a high level of approval for the healthy lifestyle nudges in the present study. The expected decline in approval rates from arguably the least (information government campaigns) to the most (default rules and choice architecture) intrusive of the three categories examined in the present study lends further evidence to current literature highlighting the role of intrusiveness [29, 30]. Specifically, differences in approval of nudges had previously been suggested to result from the degree of intrusiveness of the nudges in people’s daily routine and life [13]. As implemented in the present study, prior studies have similarly clustered nudges according to different levels of intrusiveness [13, 16]. Accordingly, non-intrusive nudges, such as providing caloric content information, received greater approval in comparison to more intrusive nudges, such as defaults and choice architectures [13, 16]. Specifically, the difference in approval for nudges belonging to different categories appears to put Singapore in the same category as many industrialised Western nations. For instance, public education campaigns were approved by 89.8% of the populace, similar to the 93% approval ratings of Canada and Australia [13]. “Traffic light” food labelling, a type of governmentally mandated nudge, was approved by 85.1% of respondents, notably more than the 55% approval rating in Japan, and closer to the 76% approval rating in Australia [13]. Finally, sweet-free cashier zones, a type of choice architecture, saw a 62.4% approval rating in Singapore, close to the 62% approval in Canada, and significantly higher than Japan (35%) and less than China (73%) [13].

Although the aforementioned results corroborate with the present findings, current literature does not yet offer much conclusive explanation for this. As noted by Arad and Rubinstein [29], a potential explanation relates to the notion that people can display a degree of psychological reactance to being nudged upon learning that they have been influenced. It is suggested that the cause for disapproval is not that an individual objects to the promoted behaviour itself, but rather to the mere fact that it externally induced rather than instigated by the person himself [30]. In relation to the present findings, this can be illustrated by comparing two nudges with different degree of intrusiveness. Classified under the category perceived to be the least intrusive, nudges such as nation-wide public education campaigns merely provide people with information to make healthier lifestyle decisions. Yet, the decision to undertake healthier choices or not ultimately lies in the individual themselves, and it is unsurprising that such autonomy corresponds to strong support for such nudges (89.8%). Conversely, nudges that are seemingly more intrusive such as imposing a tax on sugar-sweetened beverages evidently garner lesser approval (44.0%) given that such autonomy in making a healthier choice is absent. Once a tax is imposed, people are visibly left with the option of making a healthier choice given how monetary considerations have now been factored into their decision-making process. Taken together, this finding highlights a need for further elucidation on reasons underlying the approval of healthy lifestyle nudges.

### Approval rates across the population as stratified by number of chronic conditions

The development of chronic conditions often results from a wide range of unhealthy habits and behaviours, such as lack of physical activity, poor nutrition, tobacco use, and excessive alcohol consumption [31]. Correspondingly, it is reasonable to speculate that there might be potential differences regarding approval of healthy lifestyle nudges given the contrast in lifestyle habits and behaviours. Interestingly, results reported relatively similar approval ratings amongst individuals with varied number of chronic conditions across all three categories of healthy lifestyle nudges. In the context of Singapore, this could be attributed to the effectiveness of various health campaigns and initiatives introduced by the government [32, 33]. One such example is the “War on Diabetes”, which focused upon raising awareness and engaging citizens to adopt healthier habits [34]. As reported by Roystonn et al. [35], such concerted efforts are well reflected across the population; with 82.7% of Singaporean adults being able to recognise symptoms and complications of diabetes. Therein, it is plausible that given such awareness and perception pertaining to health-related issues (e.g., diabetes), majority within the population endorse health-related efforts regardless of their chronic condition status.

### Factors associated with healthy lifestyle nudges

#### Ethnicity

Prior works have examined approval rates of healthy lifestyle nudges across countries or countries comprising largely of a single ethnic group [15, 16]. Contrastingly, a key feature of the present study relates to the investigation of healthy lifestyle nudges approval rates within a multi-ethnic population. Interestingly, the present study revealed important ethnic differences for healthy lifestyle nudges approval rates across all three categories. Individuals of Malay and Others ethnicities approved more of government information campaigns as compared to those of Chinese ethnicity, while those of Malay and Indian ethnicity approved more of information governmental mandated, and default rules and choice architecture nudges than those of Chinese ethnicity. Literature examining the role of ethnicity in healthy lifestyle has highlighted significant ethnic differences in health-related aspects such as diet and exercise in Singapore [36, 37]. For example, in the National Nutrition Survey conducted in 2010, it was reported that Indians consumed the most bread and breakfast cereals, vegetable dishes, fruit, milk and dairy products, all of which are synonymous with a healthier dietary pattern. Similarly, it is possible that such health-conscious choices translate to a greater endorsement of health-related nudges. Nonetheless, ethnicity is complex and is defined by an interplay of characteristics which include spoken language, religious beliefs and common heritage, and people within and between ethnic groups can vary considerably. Therefore, caution needs to be applied in assuming the present findings are transferable.

#### Gender

As demonstrated in current literature, the present study also found a significant association between gender and healthy lifestyle nudges for the government mandated information domain. For example, Reisch et al. [38] reported that women showed greater approval rates as compared to men in nudges related to choice editing in supermarkets, subliminal advertising, sweet-free cashier zones, and meat-free day. Similarly, Reisch and Sunstein [38] also demonstrated that women favoured nudges across the domains of information government campaigns, government mandated information, default rules, manipulation and other mandates in contrast to their male counterparts. This association could be partly explained by the fact that eating, body size, and image are often a stronger concern for women than men, where such health-conscious attitudes could possibly drive the stronger approval of health-related nudges [39, 40]. Therein, the present finding is corroborated by existing literature, further highlighting a need to focus on men while planning for health related nudges.

#### Health-related behaviours

Across the population, the present study also reported significant associations between various lifestyle behaviours and approval rates of healthy lifestyle nudges. Specifically, individuals who were rated as physically active, and demonstrated better dietary patterns (in terms of DASH score) reported greater approval for government mandated information and default rules and choice architecture respectively. While the aforementioned highlights significant associations between a range of lifestyle behaviours and healthy lifestyle nudges, the overall pattern is straightforward: in general, individuals demonstrated greater support for healthy lifestyle nudges when such habits and behaviours were already reflected in their lifestyles. This is supported by widespread literature consistently demonstrating that an individual’s attitudinal approval or disapproval is associated with proclivity for healthy behaviours such as healthy diet or physical activity [41, 42]. In the context of Singapore, Banerjee and Ho [43] similarly found that Singaporeans who adopted a healthier lifestyle were also more approving of health-related campaigns and mass media messages. Given the extensive evidence of the association between attitudes and healthy lifestyle behaviours, it is unsurprising to observe similar pattern of results in the present study. Nonetheless, it should be noted that prior studies solely explored individual’s attitudes towards healthy lifestyles in general and did not focus upon the area of healthy lifestyle nudges specifically.

### Limitations

Several limitations of the present study warrant comment and conclusions drawn should be considered in light of these limitations. For instance, the lifestyle patterns of sedentary behaviour, physical activity, and dietary patterns were primarily based on self-reported recall of the respondent. In that regard, we are unable to rule out the likelihood of recall bias in this format. In the case of sedentary behaviour, a recent systematic review reported that single-item self-report measures generally underestimate sedentary time in comparison to device measures [44]. Similarly, various studies have reported that individuals tended to overestimate their levels of physical activity [45, 46]. The study did not consider some factors that have been reported to be associated with acceptance of health-nudges. In the review by Diepeveen et al. [17], such factors include familiarity (i.e., those that the public are familiar with), and engagement (i.e., acceptance is generally higher where people do not engage in the targeted behaviour themselves). Lastly, given the fact that there is no existing methodology in relation to the classification of the healthy lifestyle nudges, they are admittedly clustered based upon the interpretation of prior studies [16, 38].

## Conclusion

The study findings indicated that most nudges were generally supported by the respondents, and that most respondents were more approving of less intrusive nudges as a way of promoting healthier lifestyle choices, as is consistent with existing literature. Moreover, both ethnicity and lifestyle choices were found to be associated with the acceptance of certain types of health-nudges and should be taken into consideration by policy makers during the usage and implementation of it in public health policy. For instance, education campaigns can attempt to change beliefs of those with less healthy lifestyles, as those who attribute being overweight tend to show less support for health-related nudges [47]. As for ethnicity, key opinion leaders and grassroot workers should be engaged to shape culturally appropriate messages and connect with individuals of Chinese ethnicity. However, further research is needed especially in longitudinal cohorts to better understand the impact of nudges on the lifestyle and health outcomes of populations. Health-nudges are not novel in Singapore – efforts such as the HealthHub platform which provides people 24/7 access to their online health records and a wealth of health-related information is one of many examples in a bid to nudge the population towards better health [48]. Results from the present study highlighting the sort of nudges people are more likely to be accepting of provides up-to-date information which policymakers can utilise to modify ongoing health-nudges if necessary. In the global context, prior studies and reviews have generally reached the conclusion that if people believe that the health-nudge has legitimate goals which fits well with the interests and values of the general public, acceptance are generally high albeit exceptions when other factors such as trust in respective government is lacking. Accordingly, information from the present study may provide certain insights to international counterparts pertaining to the effectiveness of health-nudges efforts in a democratic and multi-ethnic nation like Singapore. Such information may serve as a reference for international counterparts to adapt or implement new health-nudges to better suit their respective population. Taken together, the present study provides up-to-date information on the acceptance of health-nudges in Singapore to better support the design and evaluation of such cost-effective efforts aimed at improving public health.

## Supplementary Information

**Additional file 1: Supplementary Table 1.** Results of univariate linear regressions examining the differences between groups of individuals for each outcome.

## Data Availability

The data that support the findings of this study are available from the senior author of the study (Dr. Mythily Subramaniam) but restrictions apply to the availability of these data, which are not publicly available. Data are however available from the authors upon reasonable request and with permission of the Institute of Mental Health Data Exchange Office.

## Abbreviations

BMI: Body Mass Index
CIDI: World Mental Health Composite International Diagnostic Interview
DASH: Dietary Approach to Stop Hypertension
GPAQ: Global Physical Activity Questionnaire
ITE: Institute of Technical Education
MET: Metabolic Equivalents of Tasks
SGD: Singapore Dollars
WHO: World Health Organization
