# Supplementary material for: Acceptance of healthy lifestyle nudges in the general population of Singapore
Source: BMC Public Health. 2022 Jul 5;22:1297. doi: 10.1186/s12889-022-13668-x (PMC9258182; doi:10.1186/s12889-022-13668-x)
Supplement: Supplementary file 1 — Additional file 1: Supplementary Table 1. Results of univariate linear regressions examining the differences between groups of individuals for each outcome. [file 12889_2022_13668_MOESM1_ESM.docx]

**Appendix**

Supplementary Table 1

Results of univariate linear regressions examining the differences between groups of individuals for each outcome

|  | **Information Government Campaigns** | | | | **Information Governmentally Mandated** | | | | **Default rules & choice architecture** | | | |
| --- | --- | --- | --- | --- | --- | --- | --- | --- | --- | --- | --- | --- |
|  | **Beta** | **95% CI** | | **p-value** | **Beta** | **95% CI** | | **p-value** | **Beta** | **95% CI** | | **p-value** |
| **Age groups (years)** |  |  |  |  |  |  |  |  |  |  |  |  |
| 18 to 34 (Reference) |  |  |  |  |  |  |  |  |  |  |  |  |
| 35 to 49 | 0.10 | -0.07 | 0.27 | 0.262 | -0.09 | -0.35 | 0.17 | 0.494 | 0.12 | -0.18 | 0.42 | 0.439 |
| 50 to 64 | 0.26 | 0.08 | 0.44 | **0.004** | -0.10 | -0.35 | 0.16 | 0.457 | 0.22 | -0.07 | 0.51 | 0.141 |
| 65 and above | 0.28 | 0.10 | 0.46 | **0.002** | -0.16 | -0.43 | 0.10 | 0.226 | 0.33 | 0.01 | 0.65 | **0.044** |
| **Sex** |  |  |  |  |  |  |  |  |  |  |  |  |
| Female (Reference) |  |  |  |  |  |  |  |  |  |  |  |  |
| Male | -0.02 | -0.15 | 0.11 | 0.743 | -0.35 | -0.53 | -0.16 | **< 0.001** | -0.20 | -0.42 | 0.01 | 0.067 |
| **Ethnicity** |  |  |  |  |  |  |  |  |  |  |  |  |
| Chinese (Reference) |  |  |  |  |  |  |  |  |  |  |  |  |
| Malay | 0.23 | 0.12 | 0.33 | **< 0.001** | 0.13 | -0.03 | 0.29 | 0.116 | 0.29 | 0.10 | 0.48 | **0.003** |
| Indian | 0.14 | 0.02 | 0.26 | **0.018** | 0.43 | 0.26 | 0.59 | **< 0.001** | 0.57 | 0.37 | 0.77 | **< 0.001** |
| Others | 0.22 | 0.06 | 0.39 | **0.009** | 0.28 | -0.01 | 0.57 | 0.059 | 0.39 | 0.04 | 0.73 | **0.029** |
| **Education** |  |  |  |  |  |  |  |  |  |  |  |  |
| Primary and below (Reference) |  |  |  |  |  |  |  |  |  |  |  |  |
| Secondary | -0.09 | -0.26 | 0.09 | 0.320 | -0.10 | -0.36 | 0.16 | 0.461 | -0.22 | -0.54 | 0.09 | 0.169 |
| Pre-U/Junior College | -0.17 | -0.46 | 0.12 | 0.254 | 0.34 | -0.10 | 0.78 | 0.128 | 0.01 | -0.55 | 0.56 | 0.983 |
| Vocational Institute/ITE^a^ | -0.10 | -0.34 | 0.14 | 0.406 | -0.12 | -0.51 | 0.27 | 0.541 | -0.15 | -0.57 | 0.27 | 0.480 |
| Diploma | -0.09 | -0.27 | 0.10 | 0.350 | 0.27 | 0.01 | 0.52 | **0.044** | -0.11 | -0.44 | 0.23 | 0.527 |
| Degree, professional certification, and above | -0.22 | -0.41 | -0.03 | **0.022** | 0.01 | -0.23 | 0.26 | 0.908 | -0.01 | -0.31 | 0.29 | 0.929 |
| **Marital Status** |  |  |  |  |  |  |  |  |  |  |  |  |
| Married/Cohabiting (Reference) |  |  |  |  |  |  |  |  |  |  |  |  |
| Single | -0.21 | -0.36 | -0.06 | **0.007** | -0.10 | -0.32 | 0.12 | 0.380 | -0.36 | -0.61 | -0.10 | **0.006** |
| Separated/Widowed/Divorced | 0.00 | -0.21 | 0.20 | 0.969 | 0.01 | -0.24 | 0.27 | 0.919 | -0.09 | -0.42 | 0.24 | 0.603 |
| **Employment status** |  |  |  |  |  |  |  |  |  |  |  |  |
| Employed (Reference) |  |  |  |  |  |  |  |  |  |  |  |  |
| Economically Inactive | 0.13 | 0.00 | 0.26 | 0.059 | 0.1 | -0.1 | 0.31 | 0.318 | 0.27 | 0.03 | 0.51 | **0.027** |
| Unemployment | 0.09 | -0.20 | 0.37 | 0.546 | 0.01 | -0.46 | 0.48 | 0.961 | 0.03 | -0.44 | 0.50 | 0.885 |
| **Monthly income (Personal)** |  |  |  |  |  |  |  |  |  |  |  |  |
| Below 2,000 (Reference) |  |  |  |  |  |  |  |  |  |  |  |  |
| 2,000 to 3,999 | -0.06 | -0.21 | 0.09 | 0.440 | -0.14 | -0.37 | 0.10 | 0.256 | -0.31 | -0.58 | -0.05 | **0.020** |
| 4,000 to 5,999 | -0.22 | -0.44 | 0.00 | 0.051 | -0.10 | -0.37 | 0.17 | 0.481 | -0.17 | -0.56 | 0.23 | 0.407 |
| 6,000 to 9,999 | -0.16 | -0.46 | 0.14 | 0.296 | -0.03 | -0.38 | 0.32 | 0.868 | 0.14 | -0.29 | 0.56 | 0.536 |
| 10,000 and above | -0.23 | -0.56 | 0.10 | 0.169 | 0.12 | -0.39 | 0.63 | 0.646 | 0.29 | -0.27 | 0.85 | 0.314 |
| No income | -0.09 | -0.36 | 0.17 | 0.492 | -0.13 | -0.52 | 0.26 | 0.515 | -0.15 | -0.55 | 0.25 | 0.470 |
| **BMI** |  |  |  |  |  |  |  |  |  |  |  |  |
| Normal range (Reference) |  |  |  |  |  |  |  |  |  |  |  |  |
| Underweight | -0.36 | -0.68 | -0.04 | **0.030** | -0.15 | -0.58 | 0.28 | 0.492 | -0.24 | -0.68 | 0.20 | 0.276 |
| Overweight | 0.11 | -0.04 | 0.26 | 0.141 | -0.08 | -0.29 | 0.14 | 0.492 | 0.02 | -0.24 | 0.28 | 0.878 |
| Obese | -0.07 | -0.28 | 0.15 | 0.535 | -0.28 | -0.59 | 0.03 | 0.073 | -0.21 | -0.57 | 0.16 | 0.273 |
| **Number of chronic conditions** |  |  |  |  |  |  |  |  |  |  |  |  |
| No chronic illness (Reference) |  |  |  |  |  |  |  |  |  |  |  |  |
| One chronic illness | 0.01 | -0.14 | 0.17 | 0.874 | 0.15 | -0.07 | 0.38 | 0.174 | 0.06 | -0.20 | 0.33 | 0.640 |
| At least two or more chronic illness | 0.12 | -0.02 | 0.27 | 0.099 | 0.09 | -0.13 | 0.31 | 0.446 | 0.07 | -0.19 | 0.33 | 0.586 |
| **Physical activity** |  |  |  |  |  |  |  |  |  |  |  |  |
| Sufficiently active (Reference) |  |  |  |  |  |  |  |  |  |  |  |  |
| Insufficiently active | 0.07 | -0.10 | 0.23 | 0.426 | 0.43 | 0.17 | 0.69 | **0.001** | 0.23 | -0.06 | 0.52 | 0.113 |
| **Sedentary behaviour** |  |  |  |  |  |  |  |  |  |  |  |  |
| < 7 hours/day (Reference) |  |  |  |  |  |  |  |  |  |  |  |  |
| ≥ 7 hours/day | -0.18 | -0.31 | -0.06 | **0.005** | -0.05 | -0.24 | 0.13 | 0.575 | -0.11 | -0.32 | 0.11 | 0.339 |
| **DASH Score** |  |  |  |  |  |  |  |  |  |  |  |  |
| < 16 (Reference) |  |  |  |  |  |  |  |  |  |  |  |  |
| 16 to ≤ 19 | 0.14 | -0.03 | 0.30 | 0.116 | 0.15 | -0.11 | 0.41 | 0.249 | 0.31 | 0.03 | 0.60 | **0.031** |
| 19 to ≤ 22 | 0.20 | 0.01 | 0.38 | **0.034** | 0.33 | 0.07 | 0.59 | **0.014** | 0.55 | 0.23 | 0.87 | **0.001** |
| > 22 | 0.17 | -0.01 | 0.35 | 0.061 | 0.42 | 0.19 | 0.65 | **< 0.001** | 0.78 | 0.49 | 1.06 | **< 0.001** |

^a^Institute of Technical Education
